# Lessons for future pandemics: Temporal evolution and rural-urban variations in the impacts of the COVID-19 on opioid use treatment

**DOI:** 10.1371/journal.pone.0310386

**Published:** 2024-09-13

**Authors:** Zhongyang He, Jonathan M. Heess, Travis Young, Zhen Lei

**Affiliations:** 1 Department of Energy and Mineral Engineering, The Pennsylvania State University, University Park, Pennsylvania, United States of America; 2 EMS Energy Institute, The Pennsylvania State University, University Park, Pennsylvania, United States of America; 3 Premier Inc., New York, New York, United States of America; 4 U.S. Forest Service, Evanston, Illinois, United States of America; Stanford University School of Medicine, UNITED STATES OF AMERICA

## Abstract

The COVID-19 pandemic introduced imminent and lasting impacts on the opioid crisis in the U.S., including a significant increase in opioid overdose and deaths and in use of telehealth in treatment. What lessons can we learn from the treatment transition during the pandemic that could help tackle the opioid crisis when future pandemics strike? In this paper, we conducted a phone survey with opioid treatment facilities in Pennsylvania to examine the COVID-19’s impacts on treatment facilities and individuals with opioid use disorder during the first year of the pandemic. We separated the lockdown period (Mid-March through Mid-May, 2020) from the reopening period that followed, and urban areas from rural areas, to explore temporal evolution and rural-urban variations in the COVID-19’s impacts. We found rural-urban heterogeneity in facilities’ adoption of telehealth in treatment and in challenges and risk factors faced by their clients during the lockdown period. During the reopening, telehealth was adopted by most facilities, and telehealth-related challenges became less salient; however, both rural and urban facilities reported higher relapse risks faced by their clients, citing factors more likely to be at clients’ end and related to socioeconomic stressors and mental health. Our results highlight the vitality of addressing socioeconomic and mental health challenges faced by individuals with OUD, via government policies and community interventions, when future pandemics strike. The findings also indicate the importance of maintaining facilities’ financial well-being to provide treatment services.

## Introduction

The emergence of COVID-19 led to widespread lockdown in the U.S. between March and May 2020 (during which period, the government closed schools, ordered non-essential businesses to close or significantly reduce their hours, and issued stay-at-home orders for residents), followed by reopening with a slow return to normal [1]. These disruptions in health care, social, and economic systems drastically worsened the opioid crisis in the U.S., with both imminent and lasting impacts [2–4]. Data from the CDC (Centers for Disease Control and Prevention) show that drug overdose deaths increased by 30% from 2019 to 2020, and by 16% from 2020 to 2021 [5]. The COVID-19 pandemic has also transformed opioid use treatment and spurred the adoption of telehealth in both counseling and MOUD (medication for opioid use disorder) services, in part thanks to unprecedented adjustments in federal and state regulations [6–12]. What lessons can we learn from the COVID-19 pandemic that could help tackle the opioid and drug crisis when future pandemics strike?

In this study, we conducted a phone survey of opioid treatment facilities in Pennsylvania, aiming to examine the dynamic impacts of the pandemic on treatment facilities and their clients (i.e., individuals with opioid use disorder, OUD), during the lockdown period (mid-March through mid-May 2020) versus the reopening period that followed. We also explored rural-urban variations where disparities in socioeconomic conditions and technology and broadband infrastructures (such as broadband) existed.

Our results highlight rural-urban heterogeneity in facilities’ adoption of telehealth and in challenges and risk factors faced by clients during the lockdown. However, during reopening, telehealth was adopted by most facilities, and telehealth-related challenges in treatment became less salient. Nevertheless, both rural and urban facilities reported higher relapse risks faced by their clients, and they cited risk factors more likely to be at clients’ end and related to socioeconomic and mental health conditions, highlighting the importance of addressing social, economic, and mental health challenges faced by individuals with OUD. Finally, the findings suggest the importance of facilities’ financial well-being in maintaining treatment service provision.

## Methods

Utilizing the 2020 National Directory of Drug and Alcohol Abuse Treatment Facilities (available at https://www.samhsa.gov/data/), we compiled a list of opioid treatment facilities in Pennsylvania that offered outpatient treatment services. We separated facilities into rural and urban facilities based on the counties they located. Rural and urban counties are classified based on 2013 National Center for Health Statistics (NCHS) Urban-Rural Classification Scheme code [13, 14]. We then generated a randomly permutated list of rural and urban facilities respectively, to determine the order in which we contacted facilities.

We conducted the phone survey between November 2020 and February 2021. For a facility, we called the phone number listed in the Directory during normal business hours. If the call was answered, we used a script to make a brief introduction and asked whether the respondent would like to participate in the survey, or transfer our call to someone else (e.g., facility directors) she considered to be more qualified to answer the survey. Our survey was designed to be 10–15 minutes long and answerable by the majority of facility staff, including front-desk staff [15]. If our initial phone call to a facility was not answered, we would make another attempt several days later. If our phone call was directed to a voicemail, we would leave a voicemail message asking for a callback and then wait for several days before attempting another phone call.

During the survey, we first asked a set of questions regarding the impacts of COVID-19 on a facility’s operation, including reduced business hours, staff absence and layoff, demand for services, and the facility’s financial situation. We then gathered responses on whether the facility provided counseling and MOUD services, and how they adjusted service provision. Next, we asked the respondent to comment on challenges that their clients faced in treatment and relapse risk. We asked the facility these questions for the lockdown and reopening periods separately, to explore the temporal evolution. See S1 File for the survey questionnaire.

In total, we contacted 109 treatment facilities in Pennsylvania. 15 facilities declined our interview request; 26 facilities did not give us call backs after we left voicemail messages; 47 facilities did not answer our calls and did not direct us to voice mailboxes; three facilities were not reached because their phone numbers were no longer in use; and one facility told us that the COVID-19 shut them down and there were no patients. Finally, we obtained 17 completed surveys that seemed to suffice for our study. Of the 17 surveys, nine were answered by management (such as Directors or Assistant Directors), and the other eight by front-desk staff. These 17 facilities are quite diverse in both geographic and urban-rural coverage, with 10 facilities (59%) located in eight rural counties and seven facilities (41%) in seven urban counties, with these counties being geographically dispersed across Pennsylvania.

As shown in Table 1, the rural and urban facilities in our study are similar in the number of staff, with the average being 14 and 15 respectively. Of the 10 rural facilities, all provided counseling services including both individual counseling and group meetings; however, only four provided MOUD services, with one being a certified OTP (opioid treatment program) offering both Methadone and non-Methadone (e.g., Buprenorphine) MOUD services and three being non-OPTs offering non-Methadone MOUD treatment. Of the seven urban facilities, six offered counseling, five offered non-Methadone MOUD (one facility did not offer counseling, but offered Buprenorphine and Naltrexone treatment, along with substance use education), but none is certified OTP.

**Table 1:** Characteristics of the facilities in the study.

	Rural facilities	Urban facilities
# of facilities	10	7
Average # of staff	14	15
# of facilities offering counseling	10	6
# of facilities offering methadone MOUD (i.e., OTP)	1	0
# of facilities offering non-methadone MOUD	3	5

We analyzed survey responses with Python Natural Language Toolkit [16] to identify themes and patterns from open-ended questions, and used Stata [17] to conduct statistical analysis and tests. This study was reviewed and approved by the Institutional Review Board of the Pennsylvania State University. We informed survey participants that no identifiable information, including the recording of phone calls, was collected. We acquired verbal consent from participants before proceeding to ask survey questions.

## Results

### Impacts of COVID-19 on facility staffing, service demand, and financial well-being

We first discuss our findings on the impacts of COVID-19 on staffing, demand for services, and financial well-being, during the lockdown and reopening respectively. The results are summarized in Table 2.

**Table 2:** The impact of COVID-19 on facility staffing, demand for service, and financial well-being, during the lockdown and reopening respectively.

		During lockdown	During reopening
(a) % of facilities reporting staff absence	Rural facilities	60%	40%
	Urban facilities	57%	43%
(b) % of facilities reporting staff furlough or layoff	Rural facilities	20%	0%
	Urban facilities	43%	14%
(c) % of facilities reporting reduced business hours	Rural facilities	10%	10%
	Urban facilities	29%	14%
(d) % of facilities reporting increased/same/decreased Demand for services	Rural facilities	40%/50%/10%	20%/40%/40%
	Urban facilities	43%/29%/29%	43%/43%/14%
(e) % of facilities reporting improved/same/worsened financial well-being	Rural facilities^i^	12.5%/75%/12.5%	25%/62.5%/12.5%
	Urban facilities^i^	0%/60%/40%	0%/60%/40%

Note: The percentages are based on the number of facilities that answered questions. All 10 rural and 7 urban facilities answered questions in Panels (a) through (d).

^i^ Eight rural and five rural facilities answered the question in Panel (e). The percentages in Panel (e) are calculated based on these facilities.

#### Facility staffing

During the lockdown, six of the ten rural facilities (60%) experienced staff absence (due to COVID-19 related sickness, quarantine, or anxiety), two (20%) had staff furlough or layoff, but only one facility (10%) reported a reduction in business hours. During the reopening, four rural facilities (40%) had staff absences, none had staff furlough or layoff, and one (10%) reported reduced business hours.

For the seven urban facilities, during the lockdown, four (57%) had staff absence, three (43%) had staff furlough or layoff, and two (29%) reduced business hours. During the reopening, three urban facilities (43%) had staff absences, one (14%) had staff furlough or layoff, and one (14%) reduced business hours.

Thus, the COVID-19 pandemic had considerable negative impacts on facility staffing during the lockdown, which to some extent was alleviated during the reopening. Despite staffing issues, the treatment facilities overwhelmingly maintained operation during both the lockdown and reopening. See Panels (a) through (c) in Table 2.

#### Demand for services

For the lockdown period, of the ten rural facilities, four (40%) reported increased demand, five (50%) similar demand, and one (10%) decreased demand for services, relative to the pre-pandemic level. During the reopening, two rural facilities (20%) reported higher demand, four (40%) similar demand, and another four (40%) lower demand.

Of the seven urban facilities, for the lockdown period, three (43%) reported increased demand, two (29%) similar demand, and another two (29%) decreased demand. During the reopening, three urban facilities (43%) reported higher demand, three (43%) similar demand, and one (14%) lower demand, relative to the pre-pandemic level.

Thus, a majority of the facilities reported increased or similar demand for services during the lockdown and reopening, while a minority of facilities reported decreased demand, citing such reasons as patients disliking telehealth and fewer drop-in clients from drug courts. Moreover, there seemed to be rural-urban heterogeneity in service demand among these facilities; for example, more urban facilities reported increased demand during the reopening than during the lockdown, while the converse held true for the rural facilities. See Panel (d) in Table 2.

#### Facility financial well-being

During the pandemic, facilities incurred additional expenses on PPE (personal protective equipment) & sanitization, COVID-19 testing for employees, and equipment for telehealth such as laptops [8, 18]. On the other hand, they could apply for government financial support; indeed several facilities in our study mentioned that they received federal loan relief through the CARES (Coronavirus Aid, Relief, and Economic Security) Act and financial assistance from the state government and other sources.

As shown in Panel (e) in Table 2, a minority of the facilities reported worsened financial conditions during the lockdown as compared to the pre-pandemic status, with urban facilities more likely to experience financial difficulty than rural ones (40% vs. 12.5%). Importantly, facilities’ financial well-being was highly and positively correlated with demand for services. The Goodman and Kruskal’s gamma correlation coefficient is 0.85 (p<0.001) between two ordinal variables, facilities’ financial situation (improved/same/worsened) and demand for service (increased/same/decreased). Indeed, one facility informed us that “due to lockdown a lot of relapses [happened] so [patient] numbers skyrocketed during the pandemic, so the facility saw financial gain from the pandemic.” Moreover, facilities’ financial well-being is also highly and negatively correlated with staff furlough/layoff, i.e., facilities with a better financial status were less likely to have staff furlough/layoff. The Goodman and Kruskal’s gamma is -0.90 (p<0.001) between the financial situation (improved/same/worsened) and staff furlough or layoff (yes/no). Both gamma coefficients are calculated based on 26 sets of responses from 13 facilities that answered both questions and from two periods. The conclusions still hold if the coefficients are calculated for two periods separately.

It is noteworthy that our data might depict a more optimistic picture of facilities’ operation and financial status than the reality, as closed (or struggling) facilities were unlikely (or less likely) to participate in our survey. Indeed, we encountered three facilities whose phone numbers were no longer in use and one facility whose phone message informed us of its closure. An urban facility also informed us that “other facilities in the area closed down and more clients were referred to our facility.”

### Impacts of COVID-19 on opioid treatment services

The pandemic spurred the use of telehealth in opioid treatment. When asked about adjustments in treatment during the COVID-19, the facilities mostly reported telehealth use, except for one urban facility reporting drug testing cuts and another urban facility transportation service cuts. See the results in Table 3.

**Table 3:** The impact of COVID-19 on use of telehealth in treatment during the lockdown and reopening, respectively.

		During lockdown	During opening
(a) % of facilities adopting telehealth in counseling	Rural facilities^i^	80%	90%
	Urban facilities^ii^	100%	100%
(b) % of facilities providing non-methadone MOUD that adopted telehealth in MOUD	Rural facilities^iii^	33%	100%
	Urban facilities^iv^	80%	80%
(c) % of OTP facilities that adopted telehealth in MOUD	Rural facilities^v^	0%	0%

Note: Not all facilities provided counseling or MOUD (See Table 1). The number of facilities providing counseling, methadone MOUD (i.e., OTP), or non-methadone MOUD are listed below and the percentages are calculated based on these facilities.

^i^ Ten rural facilities provided counseling services.

^ii^ Six urban facilities provided counseling services.

^iii^ Three rural facilities provided non-methadone MOUD.

^iv^ Five urban facilities were non-OTP but provided non-methadone MOUD.

^v^ One rural facility was an OTP.

### Use of telehealth in counseling

As shown in Panel (a) in Table 3, during the lockdown period, of the 10 rural facilities providing counseling, eight (80%) adopted telehealth in counseling, while the remaining two had in-person meetings in smaller groups and for shorter hours. Of the eight rural facilities that adopted telehealth in counseling during the lockdown, seven continued telehealth in counseling during the reopening, but one stopped it because “patients didn’t like it (telehealth);” meanwhile, the two rural facilities that previously did not adopt telehealth in counseling began to use it during the reopening. Two of the nine rural facilities that used telehealth in counseling during the reopening employed a hybrid model whereby patients could choose to meet online or in person.

Of the six urban facilities offering counseling services, all adopted telehealth in counseling during the lockdown and maintained it during the reopening. Three of them utilized a hybrid model.

### Use of telehealth in MOUD

During the lockdown, of the three rural facilities offering non-Methadone MOUD, one (33%) adopted telehealth. During the reopening, the rural facility that had adopted telehealth kept it; meanwhile, the two rural facilities that did not adopt telehealth during the lockdown started it. The one rural facility that was a certified OTP adopted neither telehealth nor take-home doses during the lockdown and reopening; instead, it offered more flexible services such as curbside methadone pick-up (conditional on negative drug tests).

Of the five urban facilities offering non-Methadone MOUD, during the lockdown, four (80%) adopted telehealth, and one still required in-person appointments for prescription. During the reopening, all four urban facilities that had adopted telehealth maintained it, while the facility that previously had not adopted telehealth still maintained non-adoption.

Thus, compared with the urban facilities that overwhelmingly adopted telehealth during the lockdown, the rural facilities were slower in adopting telehealth and in particular telehealth in MOUD, suggesting that rural facilities might be more conservative regarding telehealth. During the reopening, the rural facilities seemed to catch up with using telehealth, in both counseling and non-methadone MOUD, indicating that with time passing, rural facilities became more comfortable with telehealth. Indeed, two rural facilities that adopted telehealth in counseling but not in MOUD during the lockdown adopted telehealth in MOUD during reopening. See Panels (b) and (c) in Table 3.

### Challenges and relapse risk for clients during COVID-19

Finally, we asked the facilities about challenges in treatment and relapse risks faced by clients, during the lockdown and reopening, respectively. The results are summarized in Table 4.

**Table 4:** Challenges faced by treatment clients and their relapse risk during the lockdown and reopening, respectively.

		During lockdown	During reopening
(a) % of facilities reporting clients experiencing technical challenges in telehealth	Rural facilities^i^	63%	11%
	Urban facilities^ii^	29%	14%
(b) % of facilities reporting clients experiencing non-technical challenges in telehealth	Rural facilities^i^	13%	11%
	Urban facilities^ii^	57%	14%
(c) % of facilities reporting non-telehealth-related challenges	Rural facilities^iii^	40%	60%
	Urban facilities^iii^	43%	71%
(d) % of facilities reporting a higher/same/lower relapse risk for their clients	Rural facilities^iii^	80%/20%/0%	80%/20%/0%
	Urban facilities^iii^	100%/0%/0%	71%/29%/0%

Note: The number of facilities adopting telehealth in counseling or MOUD changed with region and periods. The numbers of facilities differ for different questions and are listed below, based on which the percentages are calculated. A facility might report multiple categories of challenges.

^i^ Eight rural facilities adopted telehealth during the lockdown, and nine during the reopening.

^ii^ All seven urban facilities adopted telehealth during both the lockdown and the reopening.

^iii^ All 10 rural facilities and seven urban ones answered questions in Panels (c) & (d).

### Challenges faced by clients in treatment

Facilities in the data reported three types of challenges that their clients faced in treatment, including technology issues in telehealth, non-technology issues in telehealth, and non-telehealth-related challenges. The technology issues in telehealth included a lack of internet access and equipment to connect to treatment providers and clients’ difficulty in properly using software for online meetings.

*“Some (clients) had issues being able to do telehealth. They don’t have internet connection, a computer, or a smartphone.”*

–*An administrative staff*

“*Virtual Zoom was difficult. Some patients did not have access to smartphones or laptops so technology was a barrier.*”

–*A front desk staff*

Non-technology issues in telehealth were related to a lack of face-to-face interactions (which diminished social support that clients benefited from in-person meetings) and staff’s inability to make judgments via client body language.

*“The biggest piece of addiction treatment is encouraging clients to spend time with each other outside treatment setting and not being able to do that since stuff is closed is the biggest setback for all of them [the clients].”*

–*An assistant supervisor*

“*Not having person to person, folks do not get to really talk to people the same as they do over Zoom since it’s not the same as in-person*”

*–A facility director*

Finally, non-telehealth-related challenges faced by clients in treatment included issues such as feelings of stress and anxiety, lack of transportation, temporary suspension of drug tests, unemployment, etc.

*“Mental health for clients is way worse.”*

–*A facility director*

“*Finding housing and employment are the worst now with the pandemic for clients*.”

*–A program manager*

As shown in Panels (a) through (c) in Table 4, during the lockdown period, most facilities that adopted telehealth reported telehealth-related challenges, citing both technology issues and non-technology issues. Importantly, rural facilities were more likely to report technology issues in telehealth as challenges faced by clients than urban ones. Specifically, five rural facilities (63%) reported technology issues in telehealth as challenges, but only one (13%) cited non-technology issues. In contrast, only two urban facilities (29%) reported technology issues in telehealth, while four (57%) cited non-technology issues. This contrast reflects the rural-urban gap in internet and equipment availability and digital literacy, which has been found to hinder telehealth use in rural areas before the pandemic [19, 20].

The facilities also reported non-telehealth-related challenges in treatment during the lockdown. Of the 10 rural facilities, four (40%) mentioned such challenges as patients’ noncompliance with PPE and mask-wearing guidelines (two facilities), unemployment (one facility), and lack of treatment motivation for clients on probation (one facility). Three (43%) of the seven urban facilities reported non-telehealth-related issues including lack of drug test/screening (three facilities) and clients on probation not checking in (one facility). Note that a facility could cite more than one non-telehealth-related issues faced by clients in treatment.

During the reopening, telehealth-related challenges, both technology and non-technology issues, seemed to be much alleviated, reported by just one (11%) of the nine rural facilities and one (14%) of the seven urban facilities that used telehealth. This indicates improvement in both technology accessibility and telehealth effectiveness during reopening. In particular, the COVID-19 pandemic highlighted the rural-urban digital divides including the disparity in broadband accessibility, spurring initiatives and investments to expand rural broadband access [21, 22]; for example, the Rural Digital Opportunity Fund allocated $20.4 billion in 2020 to finance high-speed broadband networks in underserved rural areas.

In contrast, non-telehealth-related challenges became more salient and diverse for clients in both rural and urban facilities during the reopening. Six (60%) of the ten rural facilities reported such issues as unemployment (three facilities), feelings of stress, anxiety, or isolation (three facilities), transportation (three facilities), housing (one facility), unable to get treatment due to self-quarantine (one facility), family issues (one facility), and less motivation in treatment (using COVID-19 exposure as an excuse to skip treatment, one facility). Five (71%) of the seven urban facilities reported challenges including unemployment (two facilities), feelings of stress, anxiety, or isolation (two facilities), transportation (one facility), housing (one facility), and lack of community meetings (one facility).

### Relapse risk

We also asked the facilities about clients’ relapse risk during the pandemic, relative to the pre-pandemic level. The results are in Panel (d) in Table 4. For the lockdown period, most facilities reported higher relapse risk, and no facilities lower risk, relative to the pre-pandemic level. Of the 10 rural facilities, eight (80%) reported increased relapse risk for clients, and the remaining two (20%) similar risks. Some risk factors cited by the rural facilities, such as fewer drug tests (two facilities), lack of in-person interaction (one facility), and medication supply (one facility), were related to facility service provision. However, more risk factors were at the clients’ end, including worsened mental health due to isolation, anxiety, and stress (three facilities), patients having more time but in boredom (two facilities), more drinking (one facility), stimulus checks (i.e., patients with additional money may be difficult to stay sober without support groups, one facility), and clients not held accountable during telehealth (one facility). Notably, one rural facility reported that during the lockdown the staff reached out to clients to maintain contact, and by so doing, clients’ relapse risk remained similar to the pre-pandemic level.

Of the seven urban facilities, all reported increased relapse risk for clients during the lockdown. The facilities cited risk factors related to facility service provisions such as fewer drug tests (one facility), as well as risk factors at clients’ end including worse mental health due to isolation, anxiety, and stress (three facilities), losing jobs (one facility), and having kids staying home from school (one facility).

*“Peer-to-peer support with social distancing, quarantine and not seeing each other, that is very difficult for them (clients)”*

–*An assistant supervisor*

*d*

“*Normally, when we have low communication with our clients it means they are more likely using*.”

–*A front desk receptionist*

“*Sometimes big influxes of money can be difficult for people in early recovery, can lead to relapse*.”

*–A facility director*

For the reopening period, most facilities (71% in urban and 80% in rural) still reported higher relapse risk than the pre-pandemic level, with no facilities reporting lower relapse risk. Risk factors for relapse during the reopening were similar to those during the lockdown. Although we asked facilities about risk factors for relapse during the lockdown and reopening separately, facilities tended to answer the question for reopening by simply saying “similar to those during the lockdown period”.

## Discussions

Our study, involving a phone survey with opioid treatment facilities in Pennsylvania, contributes to a sizable literature that examines treatment facilities and services, and individuals with OUD during the pandemic, but mostly focuses on snapshots of the impacts of the COVID-19 [23–28]. Our study instead focuses on both temporal evolution (the lockdown vs. reopening periods) and rural-urban variations regarding the COVID-19’s impacts on treatment facilities and their clients.

We find that compared to urban facilities, rural facilities seemed to be slower in adopting telehealth (and in particular telehealth in MOUD) during the lockdown, but caught up during reopening. Rural facilities were also more likely to report technology issues in telehealth as challenges faced by clients during the lockdown, while urban facilities were more likely to report non-technology issues in telehealth. However, telehealth-related challenges, both technology and non-technology issues, seemed to be less salient during the reopening period. These results not only echo the existence of rural-urban disparities in technology and broadband accessibility prior to COVID-19 [19, 22, 29–32], but also suggest telehealth-related issues seemed to be largely addressed for most facilities in our survey.

On the other hand, most facilities in our study, both rural and urban ones, cited diverse non-telehealth-related challenges faced by individuals with OUD during the reopening. They also reported higher relapse risk for clients during both the lockdown and reopening, which is consistent with the spike in opioid overdose deaths in 2020. While some risk factors cited by the facilities, such as lack of drug testing, were related to treatment service provision, most were at clients’ end, including feelings of stress, anxiety, or isolation, as well as unemployment, transportation, and housing issues [33]. Such client-end factors could be more persistent and critical in impacting opioid and drug use and overdose during future crises of pandemics.

Hence, given that the COVID-19 has spurred telehealth use in treatment and significant expansion of technology and broadband infrastructure, maintaining treatment via telehealth may cause fewer challenges for both treatment providers and clients during future pandemics, although further investment in infrastructure and subsidies for internet service may still be needed for disadvantaged communities. On the other hand, addressing social, economic, and mental health challenges faced by individuals with OUD, through government policies and community interventions, could be more crucial for tackling opioid and drug use during future pandemics [34, 35]; for example, resilient local outreach programs, including active contacting clients by treatment providers, might be particularly important in keeping individuals with OUD connected and supported.

Furthermore, we find that treatment facilities’ financial well-being was significantly and negatively associated with staff furlough/layoff during the COVID-19 pandemic, which could in turn impact treatment service provision. Since staff furlough/layoff during the COVID-19 was widely reported by facilities [8, 36, 37], with damaging consequences such as temporary suspension of new patient in-take [10, 38], it is critical to ensure facilities’ financial resilience during future pandemics. One important approach is through government funding and support. However, facilities vary in their capability to apply for government funding [37]; thus it is important to assist facilities that are smaller and/or in more disadvantaged communities in applying for and securing government funding when future pandemics strike.

Our findings also show that facilities’ financial well-being was significantly and positively correlated with demand for treatment services that could vary across facilities (for example, in our study rural facilities were more likely to experience a lower demand for treatment during the reopening than urban facilities). More research is needed to understand the heterogeneity in demand for treatment across facilities—for example, how much difference it makes to have a flexible treatment model that allows hybrids of in-person and telehealth, or to offer medication pick-up and drop-off [39–41]. Such knowledge will be helpful for treatment facilities to maintain demand for services during future pandemics.

### Limitations

There are several limitations in our study. First, the facilities in our data likely are not representative of more than 800 treatment facilities in Pennsylvania. As mentioned above, closed (or struggling) treatment facilities were unlikely (or less likely) to participate in the survey. Second, the sample size is small, and thus in many cases, it is not possible to test statistical significance in temporal evolution (the lockdown vs. reopening) and rural-urban variation for many responses. Third, the study focuses on treatment facilities in Pennsylvania, and our findings might not be generalizable to other states where socioeconomic conditions, health service sources, and technology infrastructure differ [19, 22, 42].

## Conclusions

We conducted a phone survey with 17 opioid treatment facilities in Pennsylvania between November 2020 and February 2021, to explore the COVID-19’s impacts on treatment facilities and clients in treatment. We separated the lockdown period (mid-March through mid-May 2020) from the reopening period that followed, and urban areas from rural areas, to capture both temporal evolution and rural-urban variations. The findings stress the importance of sustaining facilities’ financial well-being and addressing socioeconomic and mental health challenges faced by individuals with OUD, via government policies, community interventions, and outreach programs. While not definitely generalizable, these results could have important implications for tackling opioid and drug use and overdose when future pandemics strike.

## Supporting information

S1 File The survey questionnaire. (DOCX)
