# Supplementary material for: Lessons for future pandemics: Temporal evolution and rural-urban variations in the impacts of the COVID-19 on opioid use treatment
Source: PLoS One. 2024 Sep 13;19(9):e0310386. doi: 10.1371/journal.pone.0310386 (PMC11398672; doi:10.1371/journal.pone.0310386)
Supplement: S1 File — (DOCX) [file pone.0310386.s001.docx]

**Supporting Information for “Lessons for Future Pandemics: Temporal Evolution and Rural-urban Variations in the Impacts of the COVID-19 on Opioid Use Treatment”**

**S1 Survey questionnaire**

**Hi, my name is _______. I’m working on a survey about the impacts of COVID-19 on operation of opioid treatment facilities. This survey will take 10-15 minutes.**

**Would you like to participate?**

- No – Ask if this is a bad time etc, trying to engage them.
- Not right now – What is a better time to call you back?
- Yes – Begin questionnaire
- Transfer to others: if the transferred number is a voice mailbox, call again. If the transferred person is willing to take the survey another time. Get the name of the person.

【*If they ask why we are doing the survey, here is the answer: We hope to gain a better idea of the challenges treatment programs and patients are facing during this time, and what policies may help.* 】

**I will ask questions about the specific facility where you are working now. Any information you provide is confidential.**

【If they ask about confidentiality, here is our further explanation: *In addition to store information securely,* w*e will not use any identifiable information related to you or your program. I will not even record your name with your responses.*】

**OK, Let us start.**

1. How many staff, including treatment staff and administrative staff, did your facility have before the pandemic? (by your facility, I refer to the specific facility where you are working now) _____­­­__________

(During Lockdown Period: mid-March to mid-May)

**I would like to ask about your facility's operations DURING the lock-down period (mid-March through mid-May) when stay-at-home orders were in place because of COVID-19.**

1. Did your facility have shorter, longer, or the same business hours DURING the lock-down, *compared to before the pandemic*?

[ ] Same hours [ ] Shorter hours [ ] Longer hours [ ] *Stayed close (skip to* ***page 5****)*

1. Was your facility impacted by staff absence DURING the lockdown, due to COVID-19 illness or quarantine or staff anxiety over COVID-19?

[ ] No [ ] Yes

1. Did your facility furlough or layoff staff DURING the lockdown?

[ ] No [ ] Yes

1. Did your facility make investments in PPE, patient screening or telemedicine, in order to continue services DURING the lockdown?

[ ] No [ ] Yes

1. How was your facility’s financial situation DURING the lockdown, *compared to before the pandemic*?

[ ] Worsened [ ] Improved [ ] The same

(If interviewee is not sure about what “financial situation” means, explain to her that it is about the facility’s revenue, cost, etc.)

Why? _________________________________________________________________________

1. Did client demand for your services increase, decrease, or stay the same DURING the lockdown, *compared to before the pandemic*?

[ ] Increase [ ] Decrease [ ] The same

Could you explain why?

| Increase | Decrease | The same |
| --- | --- | --- |
| [ ] People were more likely to have OUD during lockdown | [ ] Clients’ concern about getting virus | [ ] |
| [ ] People had more free time and are more willing to get treatment | [ ] Clients’ difficulty in getting services such as medicines and counseling | [ ] |
| [ ] People like inmates that have OUD came back to communities | [ ] Clients drop out of treatment  [ ] Services, such as counseling services, became less effective |  |

*Write unlisted responses here:*

1. Did your facility provide MAT (medication assisted treatment) BEFORE the pandemic?

[ ] No [ ] Yes

(If NO) Why didn’t your facility offer MAT options? __________________________________

(IF YES) What were the main changes that your facility made to MAT services DURING the lockdown?

| [ ] Stopped the service |
| --- |
| [ ] Tele-prescribed with videos |
| [ ] Tele-prescribed with audio (telephone) |
| [ ] Switched to and/or expanding take home medication options  [ ] Switched to other MAT options such as buprenorphine, naltrexone  [ ] Admitted of new clients without initial in-person evaluation  [ ] Eliminated of prior authorization  [ ] Lengthened refill windows |

*Write unlisted responses here:*

1. Did your facility provide Individual Counseling and/or Group Therapy services BEFORE the pandemic?

[ ] No [ ] Yes

(IF YES) What were the main changes your facility made to the Counseling services DURING the lockdown?

| [ ] Stopped the service |
| --- |
| [ ] Increased teleconferencing, with videos |
| [ ] Increased teleconferencing, with audio (phones) |

Write unlisted response here:

1. Were any other services in your facility cut DURING the lockdown?

[ ] No [ ] Yes.

If Yes, what services? ___________________

1. What were the biggest challenges that your clients had in continuing treatments DURING the lock-down?
2. Do you think your clients were at higher or lower risk of relapse and overdose DURING the lock-down, *compared to before the pandemic*?

[ ] Lower [ ] Higher [ ] The same

**Why? _______________________________________________________________________**

(Now)

**Next, I would like to ask about your facility's operations NOW, during reopening.**

1. Does your facility have shorter, longer, or the same business hours NOW, *compared to before the pandemic*?

[ ] Same hours [ ] Shorter hours [ ] Longer hours [ ] Stayed close

1. **Is your facility impacted by staff absence NOW, due to COVID-19 illness or quarantine or anxiety about the virus?**

[ ] No [ ] Yes

1. **Is your facility furloughing or laying off staff NOW?**

[ ] No [ ] Yes

1. **How is your facility’s financial condition NOW, *compared to before the pandemic*?**

[ ] Worsened [ ] Improved [ ] The same

Why? _________________________________________________________________________

1. **Has client demand for services increased or decreased NOW, *compared to before the pandemic*?**

[ ] Increase [ ] Decrease [ ] The same

Could you explain why?

| Increase | Decrease |
| --- | --- |
| [ ] There are more people with opioid use disorder | [ ] With reopening clients become busier in other things |
| [ ] Clients become less concerned about getting virus | [ ] With reopening, there are fewer people with opioid use disorder |
| [ ] Clients have less difficulty in getting medicines and counseling services now than during lock-down | [ ] |
| [ ] The services, such as counseling service, are getting more effective |  |

Write unlisted response here:

1. Opioid overdoses nationwide have reportedly increased during the pandemic. Do you sense this uptick in overdoses in your facility?

[ ] No [ ] Yes

[If YES] What impacts does this uptick in opioid overdoses have on your facility?

**Only ask if they provide MAT services and made changes to MAT practices during the lock-down**

1. Has your facility NOW kept any of the adjustments you made to your MAT services during the lockdown?

[ ] No. Please explain ________________________

[ ] Yes. What are the adjustments being kept? ______________________________

**Only ask if they made changes to individual/group counseling practices during the lock-down**

1. Has your facility NOW kept any of the adjustments you made to your Individual Counseling/Group Therapy services during the lockdown?

[ ] No. Please explain _______________________________

[ ] Yes. What are the adjustments being kept? _______________________________

1. **Is your facility cutting some of your services NOW, *compared to before the pandemic*?**

[ ] No

[ ] Yes. What are the services still being cut? ______________________________

1. What are the biggest challenges your clients are faced with in continuing treatments NOW?
2. Do you think your clients are at higher or lower risk of relapse and overdose NOW, *compared to before the pandemic*?

[ ] Lower [ ] Higher [ ] The same

Why? _________________________________________________________________________

Those are all the questions I have for you today.

Would you like to give us your facility’s e-mail, so that we will send you findings from this project later on?

Do you have any questions for me?

Great! If you have any further questions or concerns, you can call us at ------------, or --------------

Thank you so much for your time!
